# Heritable Cancer Syndromes Related to the Hypoxia Pathway

**DOI:** 10.3389/fonc.2016.00068

**Published:** 2016-03-22

**Authors:** John Clark Henegan, Christian R. Gomez

**Affiliations:** ^1^Division of Hematology/Oncology, Department of Medicine, University of Mississippi Medical Center, Jackson, MS, USA; ^2^Cancer Institute, University of Mississippi Medical Center, Jackson, MS, USA; ^3^Department of Radiation Oncology, University of Mississippi Medical Center, Jackson, MS, USA; ^4^Department of Pathology, University of Mississippi Medical Center, Jackson, MS, USA

**Keywords:** von Hippel–Lindau disease, hereditary leiomyomatosis and renal cell cancer, SDHx hereditary paraganglioma–pheochromocytoma syndromes, hypoxia-inducible factor, pseudo-hypoxia

## Abstract

Families of tumor-suppressor genes, such as those involved in homologous recombination or mismatch repair, contain individual genes implicated in hereditary cancer syndromes. Collectively, such groupings establish that inactivating germline changes in genes within pathways related to genomic repair can promote carcinogenesis. The hypoxia pathway, whose activation is associated with aggressive and resistant sporadic tumors, is another pathway in which tumor-suppressor genes have been identified. von Hippel–Lindau disease, some of the hereditary paraganglioma–pheochromocytoma (PGL/PCC) syndromes, and the syndrome of hereditary leiomyomatosis and renal cell carcinoma are heritable conditions associated with genes involved or associated with the hypoxia pathway. This review links these heritable cancer syndromes to the hypoxia pathway while also comparing the relative aggression and treatment resistance of syndrome-associated tumors to similar, sporadic tumors. The reader will become aware of shared phenotypes (e.g., PGL/PCC, renal cell carcinoma) among these three hypoxia-pathway-associated heritable cancer syndromes as well as the known associations of tumor aggressiveness and treatment resistance within these pathways.

## Introduction

Heritable cancer syndromes provide important clinical and research avenues. Clinically, diagnosing a heritable cancer syndrome allows a patient and his/her family to receive appropriate, targeted cancer screenings or preventive interventions. From a research standpoint, discovery and investigation of heritable cancer syndromes allows for better understanding of mechanisms of carcinogenesis and tumor behavior.

Families of tumor-suppressor genes consist of individual genes implicated in hereditary cancer syndromes that share common molecular pathways, such as the homologous recombination (e.g., *BRCA1*, *BRCA2*, *PALB2*) or mismatch repair (e.g., *MLH1*, *MSH2*, *MSH6*) pathways. Collectively, such groupings establish that inactivating germline changes in genes within pathways related to genomic repair can promote carcinogenesis. Insights related to these pathways led to the development of pathway-related therapy (e.g., poly ADP ribose polymerase inhibitors) (1) and promising hypotheses regarding personalized, targeted therapy (e.g., PD-1 blockade in mismatch repair-deficient tumors) (2).

**Figure 1:**
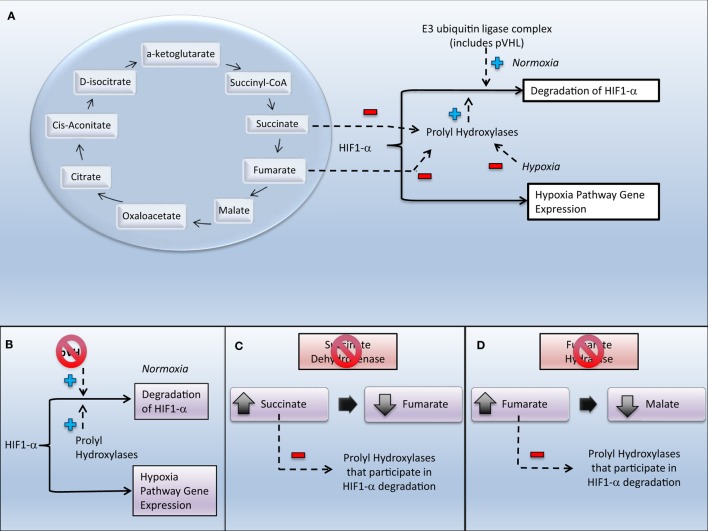
**Relationships between select heritable cancers and the hypoxia pathway**. Inactivation of von Hippel–Lindau protein (pVHL), succinate dehydrogenase (SDH), or fumarate hydratase (FH) leads to increased expression of genes in the hypoxia pathway. **(A)** Tricarboxylic acid cycle and its relationship to regulation of hypoxia-inducible factor. **(B)** Inactivation of pVHL in von Hippel–Lindau disease causes upregulation of genes expressed in the hypoxia pathway through decreased degradation of HIF1-α. **(C)** Inactivation of SDH in the SDHx hereditary paraganglioma–pheochromocytoma syndromes causes increase in succinate, which inhibits prolyl hydroxylases that would assist in the degradation of HIF. **(D)** Inactivation of FH in HLRCC causes increase in fumarate, which inhibits prolyl hydroxylases that would assist in the degradation of HIF.

Families of tumor-suppressor genes have also been identified within or affecting pathways related to the tumor microenvironment – in particular, the hypoxia pathway (Figure 1). Under normal cellular conditions, the transcription factor hypoxia-inducible factor 1 (HIF1) (3) regulates the cellular response to variations in oxygen tension. This transcription factor is a heterodimer formed by an alpha and a beta subunit. Degradation of the alpha subunit (HIF1-α) is regulated by oxygenation – when cellular oxygenation is low HIF1-α degradation is decreased, allowing HIF1 to promote cellular survival and growth (3). In malignancies, this “hypoxia driver” phenotype utilizes the hypoxia pathway to produce an aggressive and/or resistant tumor (4). Pseudo-hypoxic states are ones that display similar hypoxia-pathway gene expression but under normoxic conditions. Pseudo-hypoxia may be achieved through inactivation of tumor-suppressor genes, such as the von Hippel–Lindau (VHL) tumor suppressor, E3 ubiquitin ligase gene (*VHL*); the genes associated with the succinate dehydrogenase (SDH) complex (the SDHx genes); and the fumarate hydratase (*FH*) gene.

The purpose of this review is to highlight the grouping of heritable cancer syndromes associated with genes (i.e., *VHL*, the SDHx genes, and *FH*) in or related to the hypoxia pathway. Since these syndromes involve germline mutations associated with activation of the hypoxia pathway, and activation of this pathway may lead to aggressive and resistant sporadic tumors, this review will also compare clinical aspects of carcinogenesis, tumor growth, local/distant spread, and treatment resistance between syndrome-associated tumors and similar sporadic tumors.

## von Hippel–Lindau Disease

von Hippel–Lindau disease is an autosomal-dominant hereditary cancer syndrome involving a germline mutation in *VHL* (5). In a VHL disease registry (6), tumors with a frequency of more than 10% in VHL disease included retinal angiomas (41%), cerebellar hemangioblastomas (60%), spinal hemangioblastomas (15%), renal cell carcinomas (RCCs) (25%), and PCCs (15%). Pancreatic carcinomas, pituitary hemangioblastomas, and duodenal carcinoid tumors are described in 5% or less of patients. These frequencies are in line with other VHL disease reviews (7).

von Hippel–Lindau disease is diagnosed (6) in a patient who fulfills any one of the following four conditions: (1) two or more CNS hemangioblastomas; (2) one CNS hemangioblastoma and a disease-associated visceral tumor (i.e., RCC, PCC, pancreatic tumor or cysts, or broad ligament cystadenomas); (3) a family history of VHL disease and one of the following: (a) retinal angioma, (b) spinal or cerebellar hemangioblastoma, (c) PCC, (d) RCC, (e) or multiple renal and pancreatic cysts; or (4) a pathogenic *VHL* variant.

Clinically, VHL disease is associated with high penetrance and a shortened lifespan. VHL disease penetrance is an estimated 97% by 60 years of age (8). The three most common disease-related causes of death in VHL disease include cerebellar hemangioblastoma (48%), RCC (27%), and pancreatic carcinoma (7%) with a mean age of death of 40.9 years (6). In a review of a heritable cancer registry review, patients with VHL disease had a significantly shorter life expectancy than patients with four other heritable cancer syndromes – neurofibromatosis 1, neurofibromatosis 2, familial adenomatous polyposis, and Gorlin syndrome (9).

*VHL* is translated into von Hippel–Lindau tumor suppressor (pVHL), a hypoxia-associated protein. pVHL is a component of an intracellular multi-protein complex that also includes elongin C, elongin B, and cullin-2. This complex is an E3 ubiquitin protein ligase that, under conditions of adequate cellular oxygenation, targets HIF1-α for destruction (10) (Figure 1). VHL disease requires a mutation or in-frame deletion/insertion (11) of *VHL* that leads to loss of a functional protein. Loss of functional pVHL leads to upregulation of HIF that increases expression of various proteins (e.g., vascular endothelial growth factor (VEGF), platelet-derived growth factor, matrix metalloproteinases, and transforming growth factor-alpha) involved in cancer growth and development.

Despite VHL disease-associated tumors manifesting earlier in life than comparable sporadic ones (8), the VHL disease-associated malignancies are less aggressive in their risk of local recurrence and distant spread. Reviews of registry data indicate that patients with VHL-associated RCC have a higher primary tumor size threshold for metastatic disease, a significantly higher overall survival (12), and an increased cancer-specific survival when compared to patients with similarly sized sporadic RCC (13). Other tumors associated with VHL disease also have less relative aggressiveness in regard to disease progression or recurrence. For example, when compared to similar sporadic tumors, VHL-associated endolymphatic sac tumors are less likely to invade surrounding structures (14), VHL-associated spinal hemangioblastomas are less likely to be clinically symptomatic (15), and resected VHL-associated pancreatic neuroendocrine tumors have a significantly lower rate of recurrence than similar sporadic tumors (16).

Malignancies associated with VHL disease seem to be as responsive, if not more so, than sporadic tumors to pharmacologic interventions. In a small, single institution retrospective review of patients with VHL disease treated with first-line sunitinib for either multifocal (29%) or metastatic (71%) RCC, there was a median progression-free survival of approximately 3.5 years with 9 of 14 patients obtaining a partial response on therapy (17). For comparison, the phase 3 trial which led to sunitinib’s approval in metastatic RCC reported a median progression-free survival of 11 months and an objective response rate of 42% (18). Perhaps the potential higher response rate in VHL disease is not surprising, as a study of sporadic metastatic clear cell RCC indicated that patients with *VHL* inactivation have a higher, albeit not statistically significant different, response rate (41 versus 31%) to VEGF targeted therapy than did sporadic tumors with wild-type *VHL* (19).

In summary, VHL disease is highly penetrant and has a relatively early age of onset for its manifestations. However, VHL disease-associated tumors are less aggressive in regard to local invasion and to potential for metastatic spread as well as more responsive to therapy when compared to similar tumors.

## SDHx Hereditary Paraganglioma–Pheochromocytoma Syndromes

The hereditary paraganglioma–pheochromocytoma (PGL/PCC) syndromes are a collection of autosomal-dominant hereditary cancer syndromes. Germline mutations associated with PGL/PCC are clustered into two groups: those involved with the pseudo-hypoxic pathway and those involved in kinase signaling pathways. The former cluster includes mutations in genes related to SDH, known as the SDHx genes (20).

The SDHx hereditary PGL/PCC syndromes are relatively newly described entities that involve a mutation in *SDHA*, *SDHB*, *SDHC*, *SDHD*, or *SDHAF2*. In 2000, the first report was published of an association of one of the SDHx genes with hereditary PGL/PCC syndromes (21). Since that time, in addition to PGL/PCC, the recognized tumor spectrum among patients with a mutation in one of the SDHx genes has been expanded to also include RCC, pituitary tumors, gastrointestinal stromal tumors, and pancreatic neuroendocrine tumors (22, 23). A meta-analysis of prevalence studies found the pooled risk for malignant PGL to be 13 and 4% for *SDHB* and *SDHD* mutations, respectively (24). Penetrance may be affected by environmental oxygenation factors as patients with *SDHD* mutations who lived at lower (as opposed to higher) altitudes have less disease penetrance, have more findings of single (as opposed to multiple) tumors, and do not typically develop PCCs (25).

The diagnosis of a SDHx hereditary PGL/PCC syndrome requires finding a germline mutation in one of the SDHx genes. In clinical practice, germline genetic testing may be considered in all patients with a PGL or PCC. However, some providers may consider factors related to the probability of detecting a mutation, such as tumor location, presence of multiple tumors, age of onset, and pathological characteristics of the tumors in their decision to recommend germline molecular testing (26, 27).

The SDHx genes are involved in the structure and/or function of SDH. SDH catalyzes the conversion of succinate to fumarate in the tricarboxylic acid cycle by removing one hydrogen atom from each of the two methylene carbons of succinate and placing them in the respiratory chain (28) (Figure 1). The four subunits of SDH include two anchorage proteins (SDHD and SDHC) that are part of the mitochondrial membrane and two catalytic proteins (SDHA and SDHB) that transfer an electron to coenzyme Q. *SDHAF2* encodes a protein needed for flavination of SDHA.

Succinate’s contribution to pseudo-hypoxia has been attributed to competitive inhibition of enzymes involved in HIF1-α degradation, changes in oxidative stress, changes in energy utilization, and alterations in gene expression. The relative increase in the succinate-to-fumarate ratio is associated with succinate competitively inhibiting alpha-ketoglutarate in its binding to HIF1/2-α prolyl hydroxylases, thus preventing these enzymes from aiding in the degradation of HIF (Figure 1) (29) and leading to pseudo-hypoxia (30). PCCs with *SDHB* knockdown, like those in familial PGL/PCC, demonstrate HIF1-α stabilization despite normoxic conditions, consistent with pseudo-hypoxia (31). This has been recapitulated in tumor specimens where dysfunction of SDH due to mutations in SDHx genes leads to events consistent with pseudo-hypoxia, including mitochondrial dysfunction (32); increased expression of HIF1-α by immunohistochemistry (33); increased expression of miR-210, a key regulator of response to hypoxia (34); and increased VEGF expression (35). Other factors that may be involved in the malignant transformation, proliferation, and survival of SDHx-related tumors include an increase in reactive oxygen species, augmentation of the Warburg effect by HIF1-α, and utilization of glutamine as an energy source (29). Alterations in epigenetic regulation (36) and differential expressions of stemness may also impact the malignant potential of SDHx-mutated PGL/PCC (37).

Paragangliomas associated with *SDHB* mutations are more aggressive and resistant to treatment than sporadic PGLs. Malignant PGLs more frequently have *SDHB* mutations than do sporadic tumors (38). In a retrospective study of 34 patients undergoing primary carotid body PGL resections, there was significantly worse disease-free survival among patients with a *SDHB* mutation than among patients without a *SDHB* mutation (39). In a cohort of patients with malignant PCC/PGL, there was an association of decreased survival for those patients with a *SDHB* mutation compared to others within this cohort (40). Clinical trials (e.g., NCT02495103) are underway to explore targeted therapies for RCC associated with SDHx gene mutations.

The relatively recently discovered SDHx hereditary PGL/PCC syndromes highlight a method of carcinogenesis involving the hypoxia pathway. Pseudo-hypoxia in SDHx hereditary PGL/PCC syndrome tumors is achieved after substrate accumulation leads to competitive inhibition of an enzyme involved in degradation of HIF1-α. In contrast to VHL-associated tumors, tumors in hereditary PGL/PCC syndromes (especially those associated with *SDHB* germline mutations) behave more aggressively and are more resistant to therapy than their sporadic counterparts.

## Hereditary Leiomyomatosis and Renal Cell Cancer

Hereditary leiomyomatosis and renal cell cancer (HLRCC) is an autosomal-dominant hereditary cancer syndrome first associated with mutations in *FH* in 2002 (41). Clinically, patients with HLRCC may present with single or multiple cutaneous leiomyomata; uterine leiomyomata; and/or a RCC, which may be tubolo-papillary, collecting-duct, or papillary type 2 (42). The risk of RCC associated with HLRCC appears variable based on geography as kindreds in the United States of America and Finland, when compared to other countries, more often have multiple HLRCC-associated RCC cases (43).

Like the SDHx hereditary PGL/PCC syndromes, the diagnosis of HLRCC is made by molecular testing. Evaluation of *FH* should be considered if either there is (a) histologically confirmed multiple cutaneous leiomyomata or (b) at least two of the following: surgery required for symptomatic uterine leiomyomata before 40 years of age, type 2 papillary RCC before 40 years of age, or a first-degree relative who meets one of the above criteria (44).

There is variable expression in HLRCC, with one study reporting 87% of patients with *FH* mutations having skin leiomyomata, 96% of females having uterine leiomyomata (typically younger in age than those with sporadic tumors) (45), and 42% having RCC (46) – although a separate reviews put the risk of RCC between 15 and 20% (47). A rare manifestation of germline *FH* mutations is PCC (48).

*FH* encodes FH, the tricarboxylic acid cycle enzyme that catalyzes the conversion of fumarate to malate (49) (Figure 1). The identification of *FH* as a tumor suppressor was the second description, following the identification of the SDHx genes in hereditary PGL/PCC syndromes, of a gene translated into an intermediary metabolism enzyme also being a tumor-suppressor gene (50). HLRCC is associated with *FH* germline changes that lead to a significant reduction in FH enzyme activity (51) and an accumulation of fumarate. Like succinate, fumarate acts as a competitive inhibitor of HIF prolyl hydroxylases, causing HIF upregulation (52).

Tumor specimens from patients with HLRCC demonstrate changes consistent with *FH* inactivation and pseudo-hypoxia. Leiomyomata associated with HLRCC have large increases in fumarate consistent with levels needed to impair HIF degradation (53). Leiomyomata associated with HLRCC, compared to sporadic leiomyomata, also demonstrate higher microvessel density and increased expression of anaerobic-associated or hypoxia responsive genes (54, 55). Other mechanisms of carcinogenesis may contribute to HLRCC tumor development as cellular models and cell lines of HLRCC-associated tumors demonstrate a dependence on glycolysis (56); alterations in expression of antioxidant-response element genes (57); changes in expression of genes involved in lipid metabolism, apoptosis, and energy production/glycolysis (58); and aberrant succination (59).

Hereditary leiomyomatosis and renal cell cancer-associated RCC is aggressive in its regional and distant spread but its relative resistance or susceptibility to therapy has yet to be demonstrated. Up to 47% of HLRCC patients with RCC present with nodal or distant metastases (60), as opposed to the 33% of patients with sporadic RCC (61). Some metastatic RCC lesions in HLRCC occur despite the primary tumor being <3 cm in size, leading to the recommendation that renal masses <3 cm cannot be observed in HLRCC – a departure from the recommendation for observation of small tumors in other RCC hereditary cancer syndromes, including VHL disease (60). There is a lack of evidence to date regarding HLRCC-associated tumors’ responsiveness to therapy although clinical trials are underway to evaluate therapeutic options for patients with HLRCC-associated RCC (e.g., NCT01130519 and NCT02495103).

Hereditary leiomyomatosis and renal cell cancer shares many similarities with the SDHx hereditary PGL/PCC syndromes. Both are relatively newly discovered heritable cancer syndromes that involve a germline mutation in a tumor-suppressor gene that is translated into a tricarboxylic acid cycle enzyme. Both lead to competitive inhibition of an enzyme that in turn decreases the degradation of HIF1-α. Clinically, both are highly penetrant and can be associated with aggressive tumors.

## Discussion

Like germline mutations in genes in the homologous recombination pathway and their association with hereditary breast and ovarian cancer; or germline mutations in mismatch repair genes and their association with colorectal cancer; germline mutations in genes associated with the hypoxia pathway (e.g., *VHL*, the SDHx genes, *FH*) appear to be associated with RCC and PGL/PCC. We suggest that future research should investigate the association of these germline mutations and these clinical phenotypes to assess, for instance, if perturbations within the hypoxia pathway drive a proportion of these tumor types.

A difference in aggressiveness and resistance to therapy was seen among these three heritable cancer syndromes associated with the hypoxia pathway (Table 1). VHL disease-associated tumors seem less aggressive and more responsive to therapy compared to similar sporadic tumors. However, tumors associated with the SDHx hereditary PGL/PCC syndromes as well as with HLRCC are more aggressive and there is ongoing research into potentially effective, personalized therapies for these syndromes. More research is needed to determine if the differential aggressiveness and resistance to therapy across these three syndromes is due to other effects associated with the germline mutations, such as alterations in energy metabolism or mitochondrial function.

**Table 1:** **Aggressiveness and treatment resistance of tumors associated with heritable cancer syndromes that lead to pseudo-hypoxia compared to similar sporadic tumors**.

	von Hippel–Lindau Disease	Reference	SDHx hereditary paraganglioma/pheochromocytoma syndrome	Reference	Hereditary leiomyomatosis and renal cell cancer	Reference
Risk of local invasion	Lower	(14)	No data	NA	No data	NA
Risk of regional or distant spread	Lower	(8)	Higher	(38)	Higher	(60)
Risk of recurrence	Lower	(8, 16)	Higher	(39)	No data	NA
Resistance to standard treatment	Less resistant	(17)	No data	NA	No data	NA
Risk of death	Lower	(12, 13)	Higher	(40)	No data	NA

## Author Contributions

JH and CG both made substantial contributions to the conception and design of the work; drafted the work and revised it critically; provided final approval of the version to be published; and agree to be accountable for all aspects of the work in ensuring that questions related to the accuracy or integrity of any part of the work are appropriately investigated and resolved.

## Conflict of Interest Statement

The authors declare that the research was conducted in the absence of any commercial or financial relationships that could be construed as a potential conflict of interest.

## Abbreviations

FH, fumarate hydratase; HIF1, hypoxia-inducible factor 1; HLRCC, hereditary leiomyomatosis and renal cell cancer, PGL/PCC, paraganglioma–pheochromocytoma; RCC, renal cell carcinoma; SDH, succinate dehydrogenase; VEGF, vascular endothelial growth factor; VHL, von Hippel–Lindau.

## References

[1] Oza AM Cibula D Benzaquen AO Poole C Mathijssen RH Sonke GS Olaparib combined with chemotherapy for recurrent platinum-sensitive ovarian cancer: a randomised phase 2 trial. Lancet Oncol (2015) 16(1):87–97. 10.1016/S1470-2045(14)71135-0 25481791

[2] Le DT Uram JN Wang H Bartlett BR Kemberling H Eyring AD PD-1 blockade in tumors with mismatch-repair deficiency. (2015) 372(26):2509–20. 10.1056/NEJMoa1500596 26028255 PMC4481136

[3] Semenza GL. Oxygen homeostasis. Wiley Interdiscip Rev Syst Biol Med (2010) 2(3):336–61. 10.1002/wsbm.69 20836033

[4] Dhani N Fyles A Hedley D Milosevic M. The clinical significance of hypoxia in human cancers. Semin Nucl Med (2015) 45(2):110–21. 10.1053/j.semnuclmed.2014.11.002 25704384

[5] Barontini M Dahia PL VHL disease. Best Pract Res Clin Endocrinol Metab (2010) 24(3):401–13. 10.1016/j.beem.2010.01.002 20833332

[6] Maddock IR Moran A Maher ER Teare MD Norman A Payne SJ A genetic register for von Hippel-Lindau disease. J Med Genet (1996) 33(2):120–7. 10.1136/jmg.33.2.120 8929948 PMC1051837

[7] Lonser RR Glenn GM Walther M Chew EY Libutti SK Linehan WM von Hippel-Lindau disease. Lancet (2003) 361(9374):2059–67. 10.1016/S0140-6736(03)13643-4 12814730

[8] Maher ER Yates JR Harries R Benjamin C Harris R Moore AT Clinical features and natural history of von Hippel-Lindau disease. Q J Med (1990) 77(283):1151–63. 10.1093/qjmed/77.2.1151 2274658

[9] Wilding A Ingham SL Lalloo F Clancy T Huson SM Moran A Life expectancy in hereditary cancer predisposing diseases: an observational study. J Med Genet (2012) 49(4):264–9. 10.1136/jmedgenet-2011-100562 22362873

[10] Iliopoulos O. Molecular biology of renal cell cancer and the identification of therapeutic targets. J Clin Oncol (2006) 24(35):5593–600. 10.1200/JCO.2006.08.8948 17158545

[11] Nordstrom-O’Brien M van der Luijt RB van Rooijen E van den Ouweland AM Majoor-Krakauer DF Lolkema MP Genetic analysis of von Hippel-Lindau disease. Hum Mutat (2010) 31(5):521–37. 10.1002/humu.21219 20151405

[12] Neumann HP Bender BU Berger DP Laubenberger J Schultze-Seemann W Wetterauer U Prevalence, morphology and biology of renal cell carcinoma in von Hippel-Lindau disease compared to sporadic renal cell carcinoma. J Urol (1998) 160(4):1248–54. 10.1097/00005392-199810000-00011 9751329

[13] Kim WT Ham WS Ju HJ Lee JS Lee JS Choi YD. Clinical characteristics of renal cell carcinoma in Korean patients with von Hippel-Lindau disease compared to sporadic bilateral or multifocal renal cell carcinoma. J Korean Med Sci (2009) 24(6):1145–9. 10.3346/jkms.2009.24.6.1145 19949673 PMC2775865

[14] Nevoux J Nowak C Vellin JF Lepajolec C Sterkers O Richard S Management of endolymphatic sac tumors: sporadic cases and von Hippel-Lindau disease. Otol Neurotol (2014) 35(5):899–904. 10.1097/MAO.0000000000000299 24662627

[15] Takai K Taniguchi M Takahashi H Usui M Saito N. Comparative analysis of spinal hemangioblastomas in sporadic disease and von Hippel-Lindau syndrome. Neurol Med Chir (2010) 50(7):560–7. 10.2176/nmc.50.560 20671381

[16] de Mestier L Gaujoux S Cros J Hentic O Vullierme MP Couvelard A Long-term prognosis of resected pancreatic neuroendocrine tumors in von Hippel-Lindau disease is favorable and not influenced by small tumors left in place. Ann Surg (2015) 262(2):384–8. 10.1097/SLA.0000000000000856 25185468

[17] Roma A Maruzzo M Basso U Brunello A Zamarchi R Bezzon E First-Line sunitinib in patients with renal cell carcinoma (RCC) in von Hippel-Lindau (VHL) disease: clinical outcome and patterns of radiological response. Fam Cancer (2015) 14(2):309–16. 10.1007/s10689-014-9771-y 25391617

[18] Motzer RJ Hutson TE Tomczak P Michaelson MD Bukowski RM Rixe O Sunitinib versus interferon alfa in metastatic renal-cell carcinoma. N Engl J Med (2007) 356(2):115–24. 10.1056/NEJMoa065044 17215529

[19] Choueiri TK Vaziri SA Jaeger E Elson P Wood L Bhalla IP von Hippel-Lindau gene status and response to vascular endothelial growth factor targeted therapy for metastatic clear cell renal cell carcinoma. J Urol (2008) 180(3):860–5; discussion 5. 10.1016/j.juro.2008.05.015 18635227

[20] Lam AK. Update on paragangliomas and pheochromocytomas. Turk Patoloji Derg (2015) 31(Suppl 1):105–12. 10.5146/tjpath.2015.01318 26177321

[21] Baysal BE Ferrell RE Willett-Brozick JE Lawrence EC Myssiorek D Bosch A Mutations in SDHD, a mitochondrial complex II gene, in hereditary paraganglioma. Science (2000) 287(5454):848–51. 10.1126/science.287.5454.848 10657297

[22] Else T. 15 years of paraganglioma: pheochromocytoma, paraganglioma and genetic syndromes: a historical perspective. Endocr Relat Cancer (2015) 22(4):T147–59. 10.1530/ERC-15-0221 26273101

[23] Niemeijer ND Papathomas TG Korpershoek E de Krijger RR Oudijk L Morreau H Succinate dehydrogenase (SDH)-deficient pancreatic neuroendocrine tumor expands the SDH-related tumor spectrum. J Clin Endocrinol Metab (2015) 100(10):E1386–93. 10.1210/jc.2015-2689 26259135

[24] van Hulsteijn LT Dekkers OM Hes FJ Smit JW Corssmit EP. Risk of malignant paraganglioma in SDHB-mutation and SDHD-mutation carriers: a systematic review and meta-analysis. J Med Genet (2012) 49(12):768–76. 10.1136/jmedgenet-2012-101192 23099648

[25] Astrom K Cohen JE Willett-Brozick JE Aston CE Baysal BE. Altitude is a phenotypic modifier in hereditary paraganglioma type 1: evidence for an oxygen-sensing defect. Hum Genet (2003) 113(3):228–37. 10.1007/s00439-003-0969-6 12811540

[26] Curras-Freixes M Inglada-Perez L Mancikova V Montero-Conde C Leton R Comino-Mendez I Recommendations for somatic and germline genetic testing of single pheochromocytoma and paraganglioma based on findings from a series of 329 patients. J Med Genet (2015) 52(10):647–56. 10.1136/jmedgenet-2015-103218 26269449

[27] Erlic Z Rybicki L Peczkowska M Golcher H Kann PH Brauckhoff M Clinical predictors and algorithm for the genetic diagnosis of pheochromocytoma patients. Clin Cancer Res (2009) 15(20):6378–85. 10.1158/1078-0432.CCR-09-1237 19825962

[28] Botham KM Mayes PA Biologic Oxidation. 30 ed New York, NY: McGraw-Hill (2015).

[29] Vicha A Taieb D Pacak K. Current views on cell metabolism in SDHx-related pheochromocytoma and paraganglioma. Endocr Relat Cancer (2014) 21(3):R261–77. 10.1530/ERC-13-0398 24500761 PMC4016161

[30] Selak MA Duran RV Gottlieb E. Redox stress is not essential for the pseudo-hypoxic phenotype of succinate dehydrogenase deficient cells. Biochim Biophys Acta (2006) 1757(5–6):567–72. 10.1016/j.bbabio.2006.05.015 16797480

[31] Saito Y Ishii KA Aita Y Ikeda T Kawakami Y Shimano H Loss of SDHB elevates catecholamine synthesis and secretion depending on ROS production and HIF stabilization. Neurochem Res (2015) 40:1–11. 10.1007/s11064-015-1738-3 26620190

[32] Fitzgerald PA Adrenal medulla and paraganglia. In: Gardner DG Shoback D, editors. Greenspan’s Basic & Clinical Endocrinology. 9th ed New York, NY: McGraw-Hill (2011). p. 345–94.

[33] Burnichon N Briere JJ Libe R Vescovo L Riviere J Tissier F SDHA is a tumor suppressor gene causing paraganglioma. Hum Mol Genet (2010) 19(15):3011–20. 10.1093/hmg/ddq206 20484225 PMC2901140

[34] Tsang VH Dwight T Benn DE Meyer-Rochow GY Gill AJ Sywak M Overexpression of miR-210 is associated with SDH-related pheochromocytomas, paragangliomas, and gastrointestinal stromal tumours. Endocr Relat Cancer (2014) 21(3):415–26. 10.1530/ERC-13-0519 24623741

[35] Span PN Rao JU Oude Ophuis SB Lenders JW Sweep FC Wesseling P Overexpression of the natural antisense hypoxia-inducible factor-1alpha transcript is associated with malignant pheochromocytoma/paraganglioma. Endocr Relat Cancer (2011) 18(3):323–31. 10.1530/ERC-10-0184 21422080

[36] Hoekstra AS de Graaff MA Briaire-de Bruijn IH Ras C Seifar RM van Minderhout I Inactivation of SDH and FH cause loss of 5hmC and increased H3K9me3 in paraganglioma/pheochromocytoma and smooth muscle tumors. Oncotarget (2015) 6(36):38777–88. 10.18632/oncotarget.6091 26472283 PMC4770736

[37] Oudijk L Neuhofer CM Lichtenauer UD Papathomas TG Korpershoek E Stoop H Immunohistochemical expression of stem cell markers in pheochromocytomas/paragangliomas is associated with SDHx mutations. Eur J Endocrinol (2015) 173(1):43–52. 10.1530/EJE-14-1164 25916394

[38] Schiavi F Boedeker CC Bausch B Peczkowska M Gomez CF Strassburg T Predictors and prevalence of paraganglioma syndrome associated with mutations of the SDHC gene. JAMA (2005) 294(16):2057–63. 10.1001/jama.294.16.2057 16249420

[39] Ellis RJ Patel D Prodanov T Nilubol N Pacak K Kebebew E. The presence of SDHB mutations should modify surgical indications for carotid body paragangliomas. Ann Surg (2014) 260(1):158–62. 10.1097/SLA.0000000000000283 24169168 PMC6980248

[40] Amar L Baudin E Burnichon N Peyrard S Silvera S Bertherat J Succinate dehydrogenase B gene mutations predict survival in patients with malignant pheochromocytomas or paragangliomas. J Clin Endocrinol Metab (2007) 92(10):3822–8. 10.1210/jc.2007-0709 17652212

[41] Tomlinson IP Alam NA Rowan AJ Barclay E Jaeger EE Kelsell D Germline mutations in FH predispose to dominantly inherited uterine fibroids, skin leiomyomata and papillary renal cell cancer. Nat Genet (2002) 30(4):406–10. 10.1038/ng849 11865300

[42] Pithukpakorn M Toro JR Hereditary leiomyomatosis and renal cell cancer. 2006 Jul 31 [Updated 2015 Aug 6]. In: Pagon RA Adam MP Ardinger HH, editors. GeneReviews^®^ [Internet]. Seattle, WA: University of Washington [1993–2016]. Available from: http://www.ncbi.nlm.nih.gov/books/NBK1252/

[43] Vahteristo P Koski TA Naatsaari L Kiuru M Karhu A Herva R No evidence for a genetic modifier for renal cell cancer risk in HLRCC syndrome. Fam Cancer (2010) 9(2):245–51. 10.1007/s10689-009-9312-2 20091131

[44] Smit DL Mensenkamp AR Badeloe S Breuning MH Simon ME van Spaendonck KY Hereditary leiomyomatosis and renal cell cancer in families referred for fumarate hydratase germline mutation analysis. Clin Genet (2011) 79(1):49–59. 10.1111/j.1399-0004.2010.01486.x 20618355

[45] Sanz-Ortega J Vocke C Stratton P Linehan WM Merino MJ. Morphologic and molecular characteristics of uterine leiomyomas in hereditary leiomyomatosis and renal cancer (HLRCC) syndrome. Am J Surg Pathol (2013) 37(1):74–80. 10.1097/PAS.0b013e31825ec16f 23211287 PMC3524342

[46] Wei MH Toure O Glenn GM Pithukpakorn M Neckers L Stolle C Novel mutations in FH and expansion of the spectrum of phenotypes expressed in families with hereditary leiomyomatosis and renal cell cancer. J Med Genet (2006) 43(1):18–27. 10.1136/jmg.2005.033506 15937070 PMC2564499

[47] Menko FH Maher ER Schmidt LS Middelton LA Aittomaki K Tomlinson I Hereditary leiomyomatosis and renal cell cancer (HLRCC): renal cancer risk, surveillance and treatment. Fam Cancer (2014) 13(4):637–44. 10.1007/s10689-014-9735-2 25012257 PMC4574691

[48] Clark GR Sciacovelli M Gaude E Walsh DM Kirby G Simpson MA Germline FH mutations presenting with pheochromocytoma. J Clin Endocrinol Metab (2014) 99(10):E2046–50. 10.1210/jc.2014-1659 25004247

[49] Bender DA Mayes PA The citric acid cycle: the central pathway of carbohydrate, lipid & amino acid metabolism. In: Rodwell VW Bender DA Botham KM Kennelly PJ Weil P, editors. Harper’s Illustrated Biochemistry. 30th ed New York, NY: McGraw-Hill (2015). p. 161–7.

[50] Bayley JP Launonen V Tomlinson IP. The FH mutation database: an online database of fumarate hydratase mutations involved in the MCUL (HLRCC) tumor syndrome and congenital fumarase deficiency. BMC Med Genet (2008) 9:20. 10.1186/1471-2350-9-20 18366737 PMC2322961

[51] Gardie B Remenieras A Kattygnarath D Bombled J Lefevre S Perrier-Trudova V Novel FH mutations in families with hereditary leiomyomatosis and renal cell cancer (HLRCC) and patients with isolated type 2 papillary renal cell carcinoma. J Med Genet (2011) 48(4):226–34. 10.1136/jmg.2010.085068 21398687

[52] Isaacs JS Jung YJ Mole DR Lee S Torres-Cabala C Chung YL HIF overexpression correlates with biallelic loss of fumarate hydratase in renal cancer: novel role of fumarate in regulation of HIF stability. Cancer Cell (2005) 8(2):143–53. 10.1016/j.ccr.2005.06.017 16098467

[53] Ratcliffe PJ. Fumarate hydratase deficiency and cancer: activation of hypoxia signaling? Cancer Cell (2007) 11(4):303–5. 10.1016/j.ccr.2007.03.015 17418405

[54] Pollard P Wortham N Barclay E Alam A Elia G Manek S Evidence of increased microvessel density and activation of the hypoxia pathway in tumours from the hereditary leiomyomatosis and renal cell cancer syndrome. J Pathol (2005) 205(1):41–9. 10.1002/path.1686 15586379

[55] Catherino WH Mayers CM Mantzouris T Armstrong AY Linehan WM Segars JH. Compensatory alterations in energy homeostasis characterized in uterine tumors from hereditary leiomyomatosis and renal cell cancer. Fertil Steril (2007) 88(4 Suppl):1039–48. 10.1016/j.fertnstert.2006.11.198 17383644

[56] Ashrafian H O’Flaherty L Adam J Steeples V Chung YL East P Expression profiling in progressive stages of fumarate-hydratase deficiency: the contribution of metabolic changes to tumorigenesis. Cancer Res (2010) 70(22):9153–65. 10.1158/0008-5472.CAN-10-1949 20978192

[57] Perrier-Trudova V Huimin BW Kongpetch S Huang D Ong P LEF A Fumarate hydratase-deficient Cell Line NCCFH1 as a new in vitro model of hereditary papillary renal cell carcinoma type 2. Anticancer Res (2015) 35(12):6639–53. 26637880

[58] Yang Y Valera V Sourbier C Vocke CD Wei M Pike L A novel fumarate hydratase-deficient HLRCC kidney cancer cell line, UOK268: a model of the Warburg effect in cancer. Cancer Genet (2012) 205(7–8):377–90. 10.1016/j.cancergen.2012.05.001 22867999 PMC3415708

[59] Adam J Hatipoglu E O’Flaherty L Ternette N Sahgal N Lockstone H Renal cyst formation in Fh1-deficient mice is independent of the Hif/Phd pathway: roles for fumarate in KEAP1 succination and Nrf2 signaling. Cancer Cell (2011) 20(4):524–37. 10.1016/j.ccr.2011.09.006 22014577 PMC3202623

[60] Grubb RL III Franks ME Toro J Middelton L Choyke L Fowler S Hereditary leiomyomatosis and renal cell cancer: a syndrome associated with an aggressive form of inherited renal cancer. J Urol (2007) 177(6):2074–9; discussion 9. 10.1016/j.juro.2007.01.155 17509289

[61] Siegel RL Miller KD Jemal A. Cancer statistics, 2015. CA Cancer J Clin (2015) 65(1):5–29. 10.3322/caac.21254 25559415

